# Tonoplast- and Plasma Membrane-Localized Aquaporin-Family Transporters in Blue Hydrangea Sepals of Aluminum Hyperaccumulating Plant

**DOI:** 10.1371/journal.pone.0043189

**Published:** 2012-08-29

**Authors:** Takashi Negishi, Kenshiro Oshima, Masahira Hattori, Masatake Kanai, Shoji Mano, Mikio Nishimura, Kumi Yoshida

**Affiliations:** 1 Graduate School of Information Science, Nagoya University, Chikusa-ku, Nagoya, Aichi, Japan; 2 G-COE in Chemistry, Nagoya University, Chikusa-ku, Nagoya, Aichi, Japan; 3 Graduate School of Frontier Sciences, The University of Tokyo, Kashiwa, Chiba, Japan; 4 Department of Cell Biology, National Institute for Basic Biology, Myodaiji, Okazaki, Aichi, Japan; 5 Department of Basic Biology, School of Life Science, The Graduate University for Advanced Studies, Myodaiji, Okazaki, Aichi, Japan; RIKEN Biomass Engineering Program, Japan

## Abstract

Hydrangea (*Hydrangea macrophylla*) is tolerant of acidic soils in which toxicity generally arises from the presence of the soluble aluminum (Al) ion. When hydrangea is cultivated in acidic soil, its resulting blue sepal color is caused by the Al complex formation of anthocyanin. The concentration of vacuolar Al in blue sepal cells can reach levels in excess of approximately 15 mM, suggesting the existence of an Al-transport and/or storage system. However, until now, no Al transporter has been identified in Al hyperaccumulating plants, animals or microorganisms. To identify the transporter being responsible for Al hyperaccumulation, we prepared a cDNA library from blue sepals according to the sepal maturation stage, and then selected candidate genes using a microarray analysis and an *in silico* study. Here, we identified the vacuolar and plasma membrane-localized Al transporters genes *vacuolar Al transporter (VALT*) and *plasma membrane Al transporter 1* (*PALT1*), respectively, which are both members of the aquaporin family. The localization of each protein was confirmed by the transient co-expression of the genes. Reverse transcription-PCR and immunoblotting results indicated that *VALT* and *PALT1* are highly expressed in sepal tissue. The overexpression of *VALT* and *PALT1* in *Arabidopsis thaliana* conferred Al-tolerance and Al-sensitivity, respectively.

## Introduction

**Figure 1:**
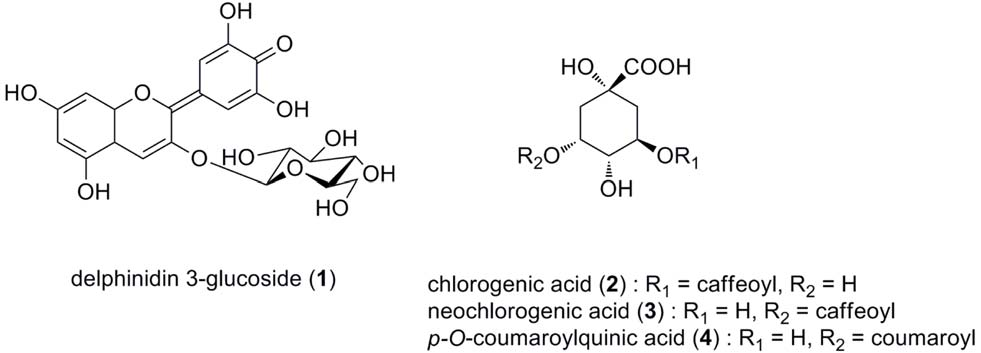
The organic components of *H. macrophylla* sepals responsible for the blue color development. All of the colored sepals contained the same anthocyanin component (delphinidin 3-glucoside, **1**) and co-pigments (chlorogenic acid, **2**; neochlorogenic acid, **3**; 5-*O-p*-coumaroylquinic acid, **4**). The sepal color is affected by the ratio of co-pigmnets, amount of Al^3+^ and vacuolar pH.

The colors of *Hydrangea macrophylla* sepals vary from blue to purple and red, and it is well known that their color easily changes with different cultivation conditions and/or with transplanting [1], [2]. The blue and red flower colors are generally developed by anthocyanins that contain different chromophores; delphinidin-series anthocyanins often appear blue, and pelargonidin- or cyanidin-series pigments appear red [3], [4]. However, all hydrangea coloration is caused by the same simple anthocyanin, delphinidin 3-glucoside (**1**) [5]–[7], in combination with the same co-pigment components, quinic acid esters (**2**;chlorogenic acid, **3**;neochlorogenic acid, **4;** 5-*O-p*-coumaroylquinic acid,) (Figure 1). The key determinant of blue sepal coloration in hydrangea is Al^3+^, and it has been established that the blue hue of sepal color increases with the increasing acidity of the soil in which hydrangea is cultivated [1], [2], [8]. It has been proposed that the blue complex of delphinidin 3-glucoside (**1**)- aluminum-neochlorogenic acid (**3**) is responsible for the blue sepal color [9], [10], but its structure has not been determined.

We have studied the chemical mechanism that is responsible for the blue coloration of hydrangea sepals by analyzing colored cells. We prepared blue and red protoplasts from each colored hydrangea sepal and reported that the blueness of the cells increased with increasing vacuolar pH [11]. Reproduction experiments of the blue and red colors by mixing cellular components with **1**–**4** (Figure 1) and Al^3+^ confirmed that the complexation of Al^3+^ with the orthodihydroxy group of the B-ring of **1** at a pH lower than 4 resulted in the formation of a water-insoluble blue pigment. However, this pigment was solubilized and stabilized by the addition of the co-pigments neochlorogenic acid (**3**) and/or 5-*O*-*p*-coumaroylquinic acid (**4**) [12]. To induce the clear development of blue color, the addition of more than 1 equivalent of Al^3+^ to **1** was required [12]. Additionally, we have quantified the concentrations of Al^3+^ in the blue and red cells as approximately 15 mM and 0.5 mM, respectively [13]. These data indicate that hydrangea accumulates high levels of Al in colored vacuoles.

Aluminum is a toxic element for plants [14], [15]. In acidic soil (less than pH 5.0), the level of water-soluble Al^3+^ increases and inhibits root growth [16]. An important task in agricultural and plant physiological research is the breeding of crops that are tolerant of acidic soil. Previous studies have primarily focused on the prevention of Al absorption by the secretion of organic acids from the root [17]–[19]. In addition to hydrangea, several plants, such as tea (*Camellia sinensis* [20], [21]) and buckwheat (*Fagopyrum esculentum* [22]) are known to be tolerant of acidic soils, and these plants accumulate Al within the plant body. However, no Al transporter gene involved in the heperaccumulation has been cloned in plants, animals, or microorganisms. Therefore, Al accumulation mechanism remains unclear.

Based on our data showing that the Al concentration in the vacuoles of blue hydrangea sepals is very high (more than 15 mM), we planned a strategy to identify transporters that are involved in Al accumulation in hydrangea. By combining the screening of a cDNA library, mRNA sequencing, microarray analyses, *in silico* studies, and Al-tolerance assays, we identified two Al-transporter genes: one encodes a tonoplast-localized transporter, and the other encodes a plasma membrane-localized transporter. We characterized those genes and discussed Al tolerance in plants.

## Results and Discussion

### The Strategy to Identify Aluminum Transporter Genes in hydrangea

To date, no aluminum transporter gene has been cloned in Al-hyperaccumulating plants nor yeast [23], [24]. The genome size of hydrangea species is approximately two billion base pairs (bp) [25], and in contrast to the model plants *Arabidopsis thaliana* and *Oryza sativa*, the complete genome has not yet been determined. Therefore, to identify Al transporter genes from *H. macrophylla*, we planned and implemented the following strategy: 1) the preparation and sequencing of a full-length cDNA library from blue sepal tissue, 2) the generation of a custom microarray that was based on these cDNAs, 3) the selection of candidate genes on a microarray and 4) the implementation of a tolerance assay using an Al-sensitive yeast strain.

**Figure 2:**
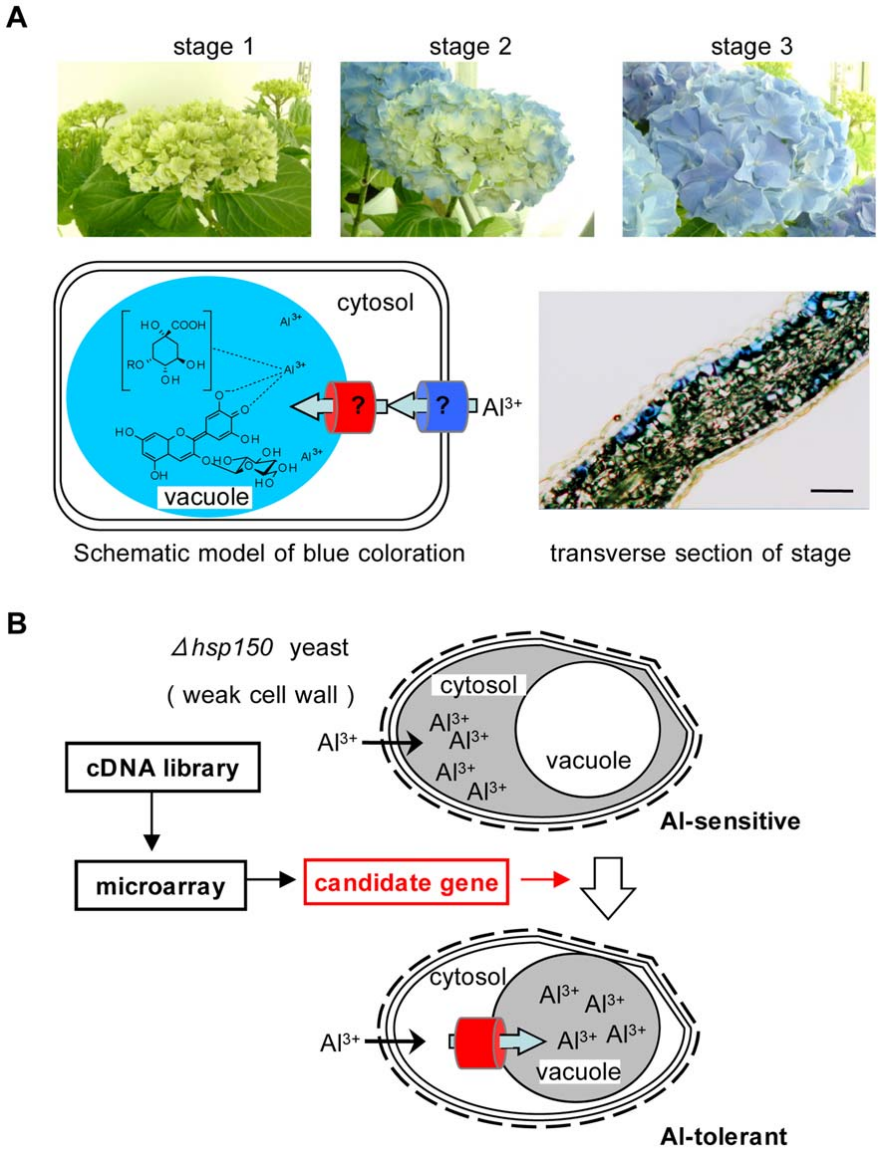
Procedure for the identification of Al transporters in hydrangea sepals. (**A**) Blue color development of *H. macrophylla* sepal. The sepals become blue during maturation, and the color was developed by an Al^3+^ complex of delphinidin 3-glucoside (**1**) and quinic acid esters (**3**, **4**) in the vacuoles of the second layer. The maturation stages were separated into three categories by its coloration as stage 1: colorless, stage 2: starting coloration and stage 3: fully colored. Scale bar of the transverse section = 50 µm. (**B**) Strategy for the identification of genes encoding a vacuolar Al transporter. The candidate genes were selected and refined by a cDNA microarray analyses and the results of database searches for their functions and subcellular localizations. The screening was conducted using *Δhsp150* yeast, which had weak cell walls and Al easily invaded into the cell. If the candidate gene encoded a vacuolar Al transport activity, this transformant in *Δhsp150* yeast should become tolerant of Al by segregating Al into vacuoles.

First, we created a normalized full-length cDNA library from blue sepal tissues at three different flowering stages (Figure 2A). One-pass sequencing of 500 clones suggested that there was no duplication of the cDNAs, and from the full sequencing of 90 clones, the average length of the cDNAs was estimated to be 1,800 bp. We completely sequenced approximately 12,000 clones to obtain their sequence tags, and we created a 12-K custom microarray (Combmatrix) from the unique sequences. This microarray was used to analyze the gene expression patterns with respect to the stages of the coloration of the sepals (GEO accession numbers GSE26665 and GPL11530). To evaluate the reliability of the gene expression in this microarray, the genes encompassing the steps from chalcone synthase (*CHS*) to anthocyanidin 3-*O*-glucosyltransferase (*3GT*) in the anthocyanin biosynthetic pathway were analyzed, and the expected expression levels were determined for almost all of these genes, with the exception of chalcone isomerase (*CHI*) and flavanone 3-hydroxylase (*F3H*).

We have previously reported that the Al content in the blue sepal tissue increases with aging; the content at stage 1 was 88 ppm, and it increased to 272 ppm at stage 3 [26]. Therefore, we hypothesized that the expression of the Al transporter gene(s) may increase during the maturation; alternatively, it should at least maintain a steady-state level. We compared the ratios of gene expression between the tissues from each stage to narrow the list of Al transporter gene candidates (Table S1). We selected candidate genes whose ratios of gene expression at stage 3/stage 1 and stage 3/stage 2 were greater than 2.0. The functions of these candidates were collated with the databases, and the possibility of membrane subcellular localization, especially of the tonoplast, was predicted using SOSUI [27] to yield 6 candidate genes, which were named *Al1*-*6*.

To identify the function of the candidate genes *Al1*-*6*, we needed to develop an efficient assay system. For this purpose, we selected the *delta heat shock protein 150* (*Δhsp150*) yeast mutant. Because the Hsp150 protein plays a role in the stability of the cell wall structure, the *Δhsp150* mutant has some deficiency in cell wall barrier; therefore, Al^3+^ may easily enter the cytosol of these cells, which induces Al sensitivity for mutants that are cultivated on a high-Al medium [28]. If a candidate gene encodes a vacuolar Al transporter, the transformed yeast could sequestrate Al^3+^ into the vacuoles, and this would be demonstrated by its survival on high-Al medium (Figure 2B).

### Identification of the Vacuolar Membrane-Localized Al Transporter

**Figure 3:**
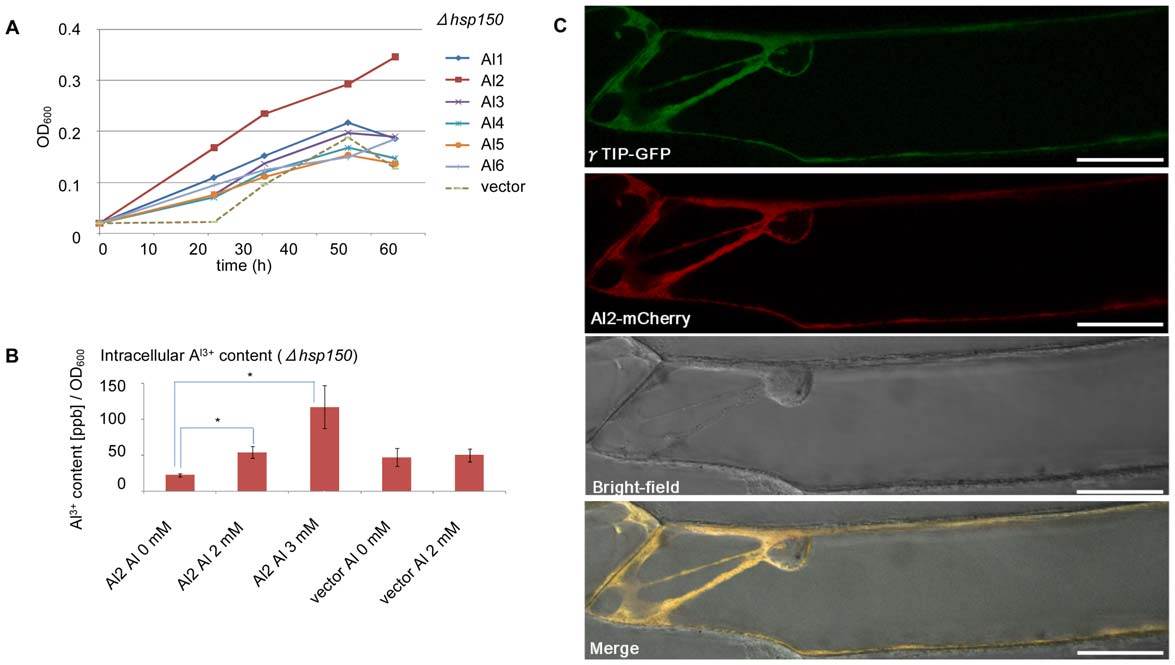
Identification of the gene of a vacuolar aluminum transporter. (**A**) Al tolerance assay of *Δhsp150* yeast cells that were transformed with the candidate genes *Al1–6.* Each cell was grown on 2 mM Al_2_(SO_4_)_3_ at pH 3.5. The time course of growth of each transformant was measured by OD_600_ for 2 d. (**B**) The intracellular Al content of the *Al2*-transformed *Δhsp150* yeast cells. The cells were exposed to different concentrations of Al_2_(SO_4_)_3_ at pH 3.5 for 2 d. The Al content of cells that carried *Al2* significantly increased with the increasing Al concentration in the medium (*P<0.05 by Student's *t* test). The error bars represent SE (n = 3). (**C**) Subcellular localization of HmVALT(Al2). *HmVALT*(*Al2*)-*mCherry* and *γTIP*-*GFP*, which was used as a control, were simultaneously introduced into onion epidermal cells by particle bombardment. The merged image shows that HmVALT is localized to the tonoplast. Scale bar = 50 µm.

The Al-tolerance assay was performed among the 6 genes (*Al1-6*) that were introduced into *Δhsp150* cells, and the growth was measured in low-pH, low-phosphate (LPP) liquid medium that contained 2 mM Al_2_(SO_4_)_3_ at pH 3.5 for 2 d. As a result, the *Al2* gene conferred Al tolerance to the transformed yeast (Figure 3A). To determine whether the *Al2* gene product specifically functions as a vacuolar Al transporter rather than secreting Al out of the cell and/or increasing the binding of Al to the cell wall, the intracellular Al content was quantified using cell-wall-digested yeast (Figure 3B). The Al content of the *Al2* transformants increased significantly from 22 ppb/OD_600_ [0 mM Al_2_(SO_4_)_3_] to 116 ppb/OD_600_ [3 mM Al_2_(SO_4_)_3_], although no increase was observed in case of the empty vector control (Figure 3B). With an increased aluminum concentration in the medium, the cells that were transformed with *Al2* could grow uninhibited in contrast to the vector control. In the vector control, the cells could not grow on the medium that contained 3 mM Al_2_(SO_4_)_3_, and the Al content of these cells could not be measured. These results strongly suggest that *Al2* is a vacuolar Al-transporter gene.

To verify the subcellular localization of the Al2 protein, a transient co-expression analysis was performed in onion epidermal cells. *Al2* was fused to *mCherry* and was simultaneously transiently expressed with the *gamma* tonoplast intrinsic protein (*γ*TIP)-green fluorescent protein (GFP) fusion; the latter was used as a marker for the vacuolar membrane [29]. The red signals from Al2-mCherry co-localized with the green signals from γTIP-GFP, which indicated that the Al2 protein was localized to the tonoplast (Figure 3C).

**Figure 4:**
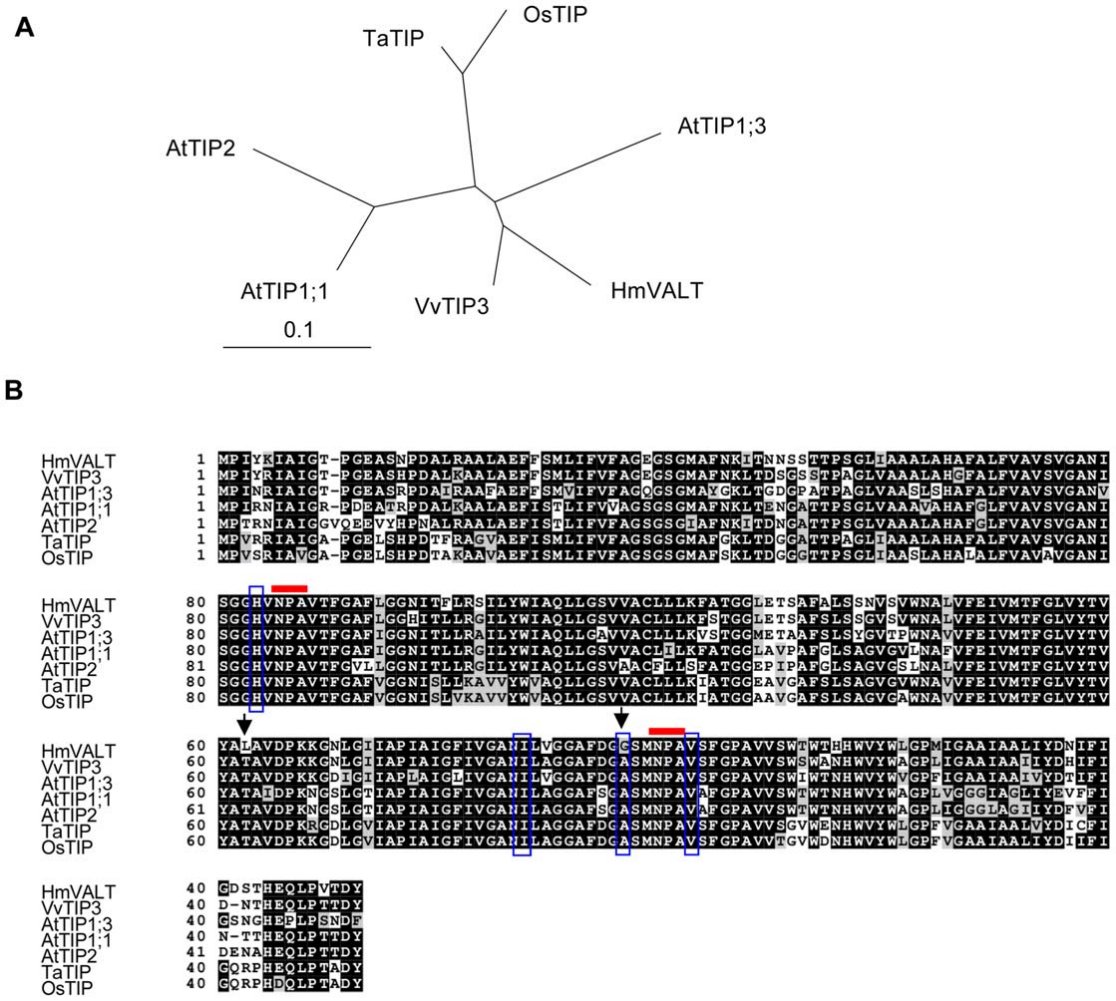
Comparison of HmVALT and the TIP proteins. (**A**) Phylogenetic tree was generated from a ClustalW alignment of HmVALT and selected TIP amino acid sequences using TreeView 1.6.6. (**B**) Alignment of the HmVALT and similar amino acid sequences in Figure 4A. Black and gray boxes indicate identical and similar amino acids, respectively. The red bars above the alignment denote the positions of the NPA motif. The aligned ar/R selectivity filter residues are highlighted in vertical blue boxes. The positions that are indicated by black arrows were mutated in the analysis of the yeast Al-sensitivity test (Figure 8). Sequences acquired from accessions: VvTIP3 [AAF78757], AtTIP1;3 [NP192056], AtTIP1;1 [NP_181221], AtTIP1;1 [NP_189283], TaTIP [ABI96817], OsTIP [NP_001045562].

We renamed the *Al2* gene as *HmVALT*, which stands for *H. macrophylla* vacuolar aluminum transporter. *HmVALT* encodes a polypeptide of 252 amino acids with two Asparagine-Proline-Alanine (NPA) motifs, whose product is a member of the TIP family that belongs to the vacuole-localized aquaporin family (Figure 4) [30]. The similarities of amino acid sequences among HmVALT, TIP3 (*Vitis berlandieri*×*Vitis rupestris*) and AtTIP1;3 were 88% and 79%, respectively. The TIP family was first identified as a water channel protein, but several aquaporin family proteins were recently found to transport molecules that are larger than water, such as NH_3_ [31], [32], H_2_O_2_ [33] and urea [34]. Here, we provide that a TIP-family protein, HmVALT, should transport aluminum. Aquaporin family proteins are recognized to transport non-ionic substrate. The chemical form of aluminum that is transported across the tonoplast may be Al(OH)_3_ because the pH of the cytosol is approximately 7.5 [16], although the evidence for this is the next problem to be elucidated.

### Identification of the Plasma Membrane-Localized Aluminum Transporter

**Figure 5:**
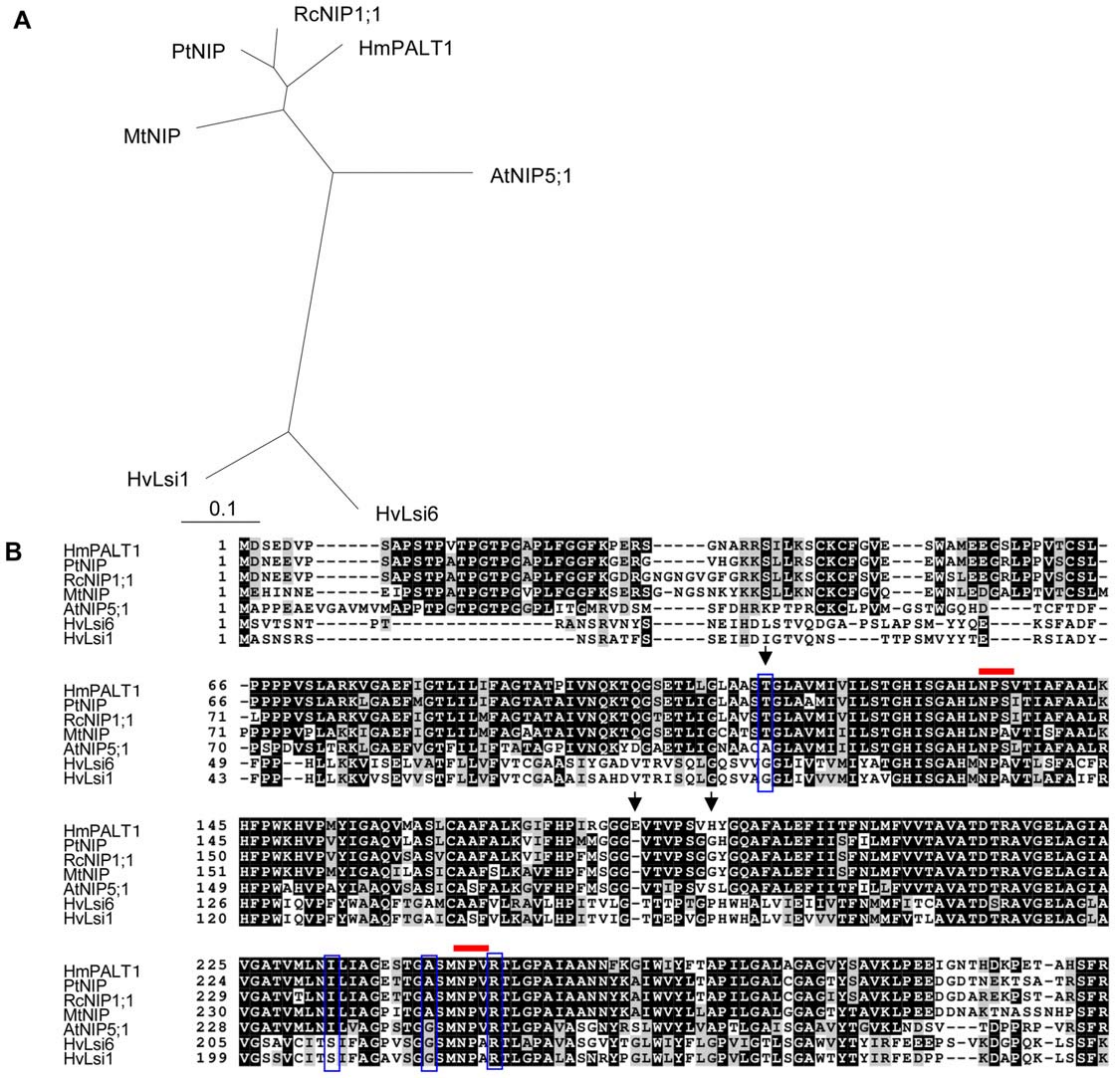
Comparison of HmPALT1 and the NIP proteins. (**A**) Phylogenetic tree was generated from a ClustalW alignment of selected NIP and HmPALT1 amino acid sequences using TreeView 1.6.6. (**B**) Alignment of HmPALT1 and similar amino acid sequences in Figure5A. Black and gray boxes indicate identical and similar amino acids, respectively. The red bars above the alignment denote the positions of the NPA motif. The aligned ar/R selectivity filter residues are highlighted in vertical blue boxes. The positions that are indicated by black arrows were mutated in the analysis of the yeast Al-sensitivity test (Figure 8). Sequences acquired from accessions: PtNIP [XP_002297797], RcNIP1;1 [XP_002527308], MtNIP [AAS48063], AtNIP5;1 [NP_192776], HvLsi6 [BAH84977], HvLsi1 [BAH24163].

To facilitate Al hyperaccumulation into the vacuoles, a plasma membrane-localized Al transporter (PM Al transporter) should be present simultaneously. Therefore, we next attempted to isolate the putative PM Al-transporter gene. From the results of the microarray analysis and genetic information, we sought to identify any aquaporin-family genes that were predicted to be localized to the plasma membrane, and that displayed increased expression with sepal maturation. We identified one gene, which is designated *HmPALT1,* whose product is 304 amino acids in length and was similar to other nodulin 26-like intrinsic proteins (NIPs); HmPALT1 was 84% similar to NIP (*Populus trichocarpa*) and 82% similar to NIP1;1 (*Ricinus communis*) (Figure 5).

**Figure 6:**
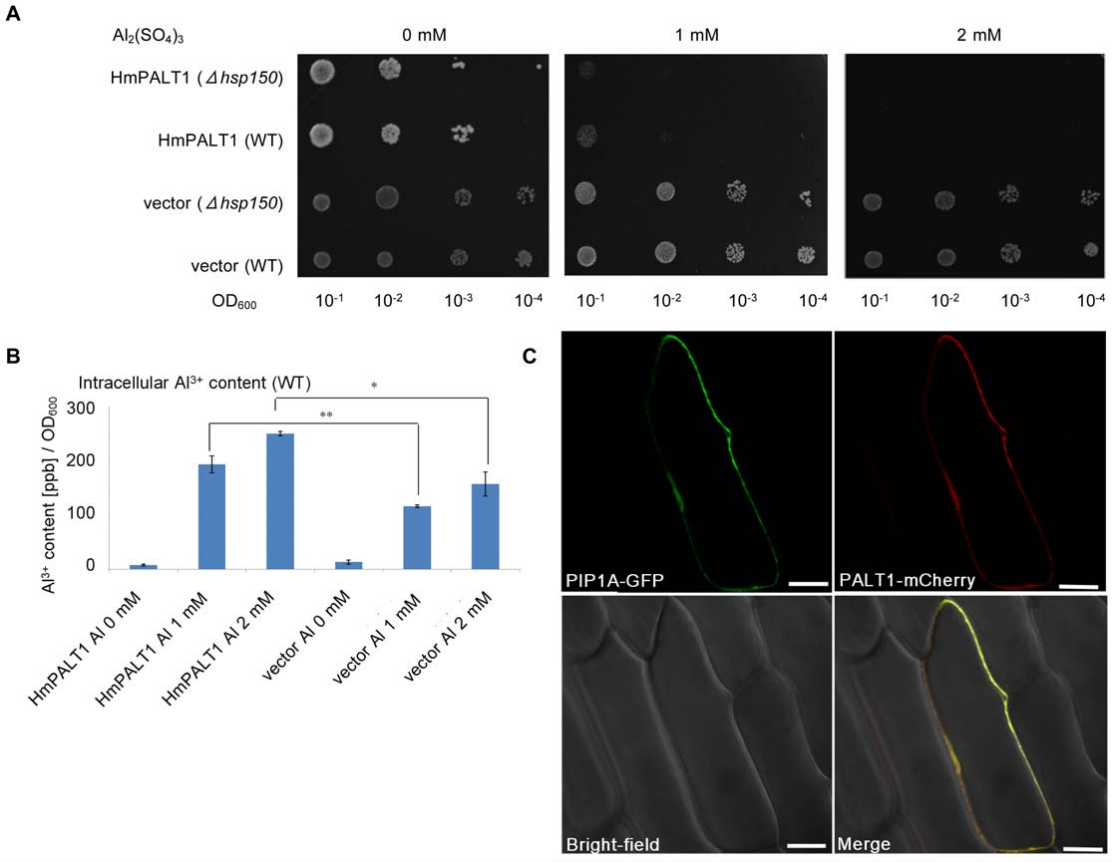
Identification of a plasma membrane-localized Al-transporter, HmPALT1, in hydrangea sepals. (**A**) Al tolerance assay of HmPALT1. *Δhsp150* and wild-type (WT) yeast cells carrying *HmPALT1* or the empty vector (pYES2) were spotted onto LPP–uracil medium (pH 3.5) with or without 1 and 2 mM Al_2_(SO_4_)_3_, and the plates were incubated at 30°C for 4 d. (**B**) The intracellular Al content of yeast cells that were transformed with *HmPALT1*. Yeast cells carrying *HmPALT1* or the empty vector were exposed to different concentrations of Al_2_(SO_4_)_3_ at pH 3.5 for 2 d. The intracellular Al content in both transformants increased with the Al in the medium in a dose-dependent manner. It is noteworthy, however, that the Al content of the yeast carrying *HmPALT1* was approximately twice as much as that of the vector control cells, which was significant (**P<0.01 and *P<0.05 by Student's *t* test, respectively). The error bars represent SE (n = 3). (**C**) Subcellular localization of HmPALT1. The construct *pHmPALT1-mCherry* was simultaneously introduced into Welsh onion epidermal cells by particle bombardment with *pPIP1A-GFP* as a plasma membrane marker. The fluorescent signals were observed under microscopy 21 h after the bombardment. The merged image shows that HmPALT1 localizes to the plasma membrane. Scale bar = 50 µm.

The Al transport activity of HmPALT1 was assayed by Al-tolerance experiments. *HmPALT1* was introduced into both *Δhsp150* and wild-type (WT) yeast cells, and each transformant was grown on LPP plates that contained 1 to 2 mM Al_2_(SO_4_)_3_ (Figure 6A). If the HmPALT1 protein was localized on the plasma membrane and functioned as an Al transporter, the Al content in the cytosol increased, then, the mutant should become more Al-sensitive. As shown Figure 6A, both of the transformants became more sensitive to Al, which suggests that the *HmPALT1* gene product may function as a PM Al transporter. To confirm this result, the intracellular Al content of the *HmPALT1*-transformed yeast cells was analyzed. The Al content in the yeast showed a dose-dependent increase from 195 ppb/OD_600_ [1 mM Al_2_(SO_4_)_3_] to 250 ppb/OD_600_ [2 mM Al_2_(SO_4_)_3_], and it was significantly different when compared with that of the vector controls (Figure 6B). These data indicated the identification of another aquaporin-family Al transporter that localized to the plasma membrane. The subcellular localization of HmPALT1 was analyzed using a fusion of HmPALT1 with mCherry and a fusion of GFP with *Arabidopsis thaliana* plasma membrane intrinsic protein 1A (AtPIP1A) [35], which served as a plasma membrane-localized protein marker (Figure 6C). The signal from HmPALT1-mCherry was merged with that of AHA2-GFP, which showed that HmPALT1 localized to the plasma membrane.

Several NIP-family [36] transporters have been reported to be involved in the transport of B [37], [38], As [39] and Si [40]. The substrate specificity of aquaporin family proteins is controlled by the pore size, which is primarily determined by the pair of NPA motifs and the aromatic/Arginine (ar/R) constriction. The latter is comprised of four residues; one residue each belongs to helix 2 (H2) and helix 5 (H5), and the other two residues belong to loop E (LE1 and the invariant R). NIP family proteins in plants have been categorized to subgroup I (W V A R), II (A/T I/A G/A R) and III (G S G R) based on this construction [41], [42]. HmPALT1 is a plasma membrane-localized Al transporter in the NIP family [36] and belongs to NIP subgroup II (Figure 5). Recently, a plasma membrane-localized Al transporter (Nrat1) belonging to the natural resistance-associated macrophage protein family in rice was reported [43]. It has been suggested that rice utilizes Al exclusion from symplast for Al tolerance mediated by the structure of cell walls and/or organic acid exudation [44], [45]. Moreover, rice is not an Al hyperaccmulator plant. Therefore, the Al transport activity of Nrat1 might not be so high. Based on these points, HmPALT1 that was isolated from the blue hydrangea sepal plays a much different role in transporting aluminum than Nrat1.

### Expression of HmVALT and HmPALT1 in Hydrangea

**Figure 7:**
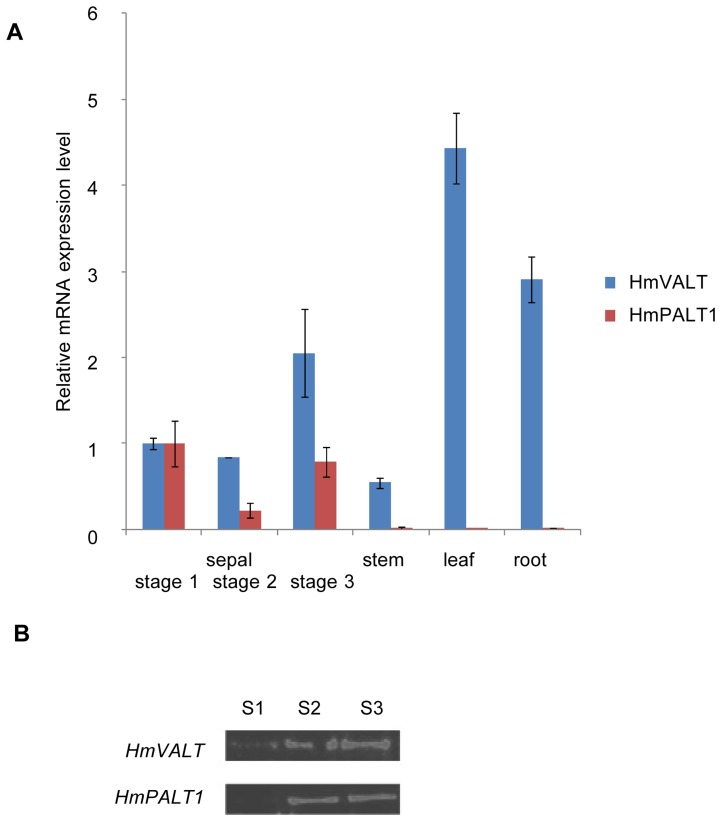
Expression of *HmVALT* and *HmPALT*1 and accumulation of their gene products in hydrangea. (**A**) The amount of *HmVALT* and *HmPALT1* mRNA was determined by quantitative RT-PCR. mRNAs were prepared from sepal at each stage and other tissues. The data are relative to the expression of 18S ribosomal RNA and were further normalized to the level of sepal at stage 1 mRNA, which was expressed as 1.0. The error bars represent SD (n = 3). (**B**) Immunoblot analyses of HmVALT and HmPALT1 in the sepals. Hydrophobic protein fractions were extracted from sepal tissues at each stage and subjected to SDS–PAGE (20 µg of protein).

We examined the stage- and tissue-specific gene expression levels of *HmVALT* and *HmPALT1* in hydrangea using a quantitative reverse transcription-PCR (qRT-PCR) analysis (Figure 7A). In sepals, both *HmVALT* and *HmPALT1* mRNAs were expressed during all of the growing stages: from the first colorless stage (stage 1) until the full colored stage (stage 3). This expression pattern can give a rational explanation why the Al accumulation in the sepal tissue was observed from an early stage and increased with maturation [26]. *HmVALT* were expressed together in the roots, stems and leaves, although *HmPALT1* were not detected in these tissues (Figure 7A). These results indicate that the set of plasma membrane- and vacuolar membrane-localized Al transporters may act coordinately to facilitate the transport and storage of Al in vacuoles in sepal. In addition, the existence of another plasma membrane-localized Al transporter in stem, leaf and root might be indicated.

The accumulation of HmVALT and HmPALT1 protein in the sepals was investigated by immunoblotting. Both proteins were detected at all stages, and increase in protein levels during sepal maturation was observed (Figure 7B). These data supported that HmVALT and HmPALT1 is responsible for hyper accumulation of Al in blue sepal vacuoles.

Concerning with the accumulation of other toxic metals in plants, the hyperaccumulation of zinc in *Arabidopsis halleri* and *Noccaea caerulescens* was reported and the mechanisms were clarified. In these plants, Zn transporters and/or Zn-chelator biosynthestic proteins were constitutively expressed [46]. Transcriptomic analysis of *A. halleri* and *N. caerulescens* with non-accumulator species revealed that a gene set encoding metal transporters and metal-chelator biosynthestic proteins was highly and constitutively expressed in the hyperaccumulators. In these hyperaccumulator plants, many proteins work simultaneously; Zrt-Irt-like protein (ZIP) transporters function in cellular uptake, P-type ATPases (Heavy Metal ATPAse 4, HMA4) function in xylem loading/unloading and Metal tolerance protein 1 (MTP1) functions in vacuolar storage [46]. To maintain their enhanced function, the aforementioned genes were constitutively expressed in plants. The constitutively high expression of the *HmVALT* and *HmPALT1* genes in hydrangea is similar to that found in Zn-hyperaccumulator plants, and this might be important for continuous Al-accumulation, storage and tolerance in hydrangea.

### Mutagenesis Analysis of HmVALT and HmPALT1 in Yeast

**Figure 8:**
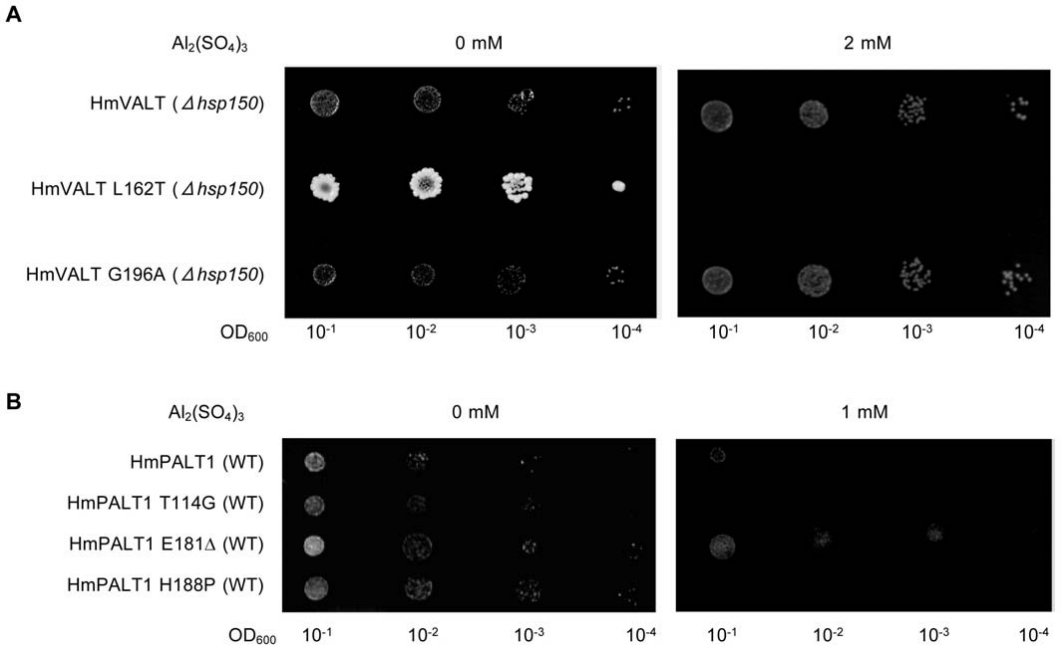
Determination of amino acids responsible for Al transport activity of HmVALT1 or HmPALT1. (**A**) Al tolerance assay of amino acid replaced HmVALT transformant. *Δhsp150* yeast cells that harbored HmVALT-L162T and −G196A were spotted onto LPP medium (pH 3.5) with or without 2 mM Al_2_(SO_4_)_3_, and the plates were incubated at 30°C for 4 d.HmVALT-L162T transformant grew less indicating to become more Al-sensitive but that of HmVALT-G196A grew at similar level. (**B**) Al tolerance assay of amino acid replaced HmPALT transformant. Wild type yeast cells that harbored HmPALT1-T114G, −E181Δ and −H188P were spotted onto LPP medium (pH 3.5) with or without 1 mM Al_2_(SO_4_)_3_, and the plates were incubated at 30°C for 4 d. HmPALT1-E181Δ transformant was less sensitive to Al, but others showed to be more sensitive.

Several distinct differences were observed in amino acid sequences of HmVALT and HmPALT1 which sites are conserved in other TIP and NIP proteins, respectively, (Figure 4, 5). To identify the key amino acid residue(s) of HmVALT and HmPALT1 being responsible for aluminum transport activity, a mutation analysis in yeast cells was performed by assaying the change of Al tolerance (Figure 8). In HmVALT 162 L and 196G (arrows in Figure 4) were presumed to be the candidate. Therefore, the substitutions of L162T (from 162th leucine to threonine) and G196A (from 196th alanine to glycine) were analyzed using *Δhsp150* cells (Figure 8A). The transformants were cultivated on Al-containing medium and as shown in Figure 8A the growth of L162T in HmVALT were inhibited, but G196A showed any difference in growth compared with 0 mM and 2 mM Al. These results indicate that 162th leucine may be important to transport Al.

In HmPALT1 114G, 181E and 188P (arrows in Figure 5) were selected to be the candidates. The substitutions of T114G (from 114th threonine to glycine), E181Δ (deletion of 181st gulutamic acid) and H118P (from 118th histidine to proline) were analyzed using wild type yeast-cells (Figure 8B). The E181Δ became more tolerant in 1 mM Al medium and other transformants did not grown in this medium. Therefore, the insertion of the 181st glutamic acid should play an important role to transport Al.

The leucine residue of HmVALT is located in the extracellular loop C [47] and is unique to HmVALT; all of the other TIPs have threonine in that site (Figure 4B). The loop C of aquaporin is presumed to facilitate the substrate selectivity [48]; therefore, the change of L162T may cause a conformational change that is followed by the decrease of aluminum selectivity and aluminum transport activity. The 181st glutamic acid (E181) in loop D of HmPALT1 is also a unique insertion in comparison with other NIPs (Figure 5B). Because loop D is predicted to be involved in aquaporin gating [47], this insertion of glutamic acid should change an electrostatic state and hydrogen bond of this loop, which may affect aluminum flow (Figure 8B). Hydrangea is famous for its requirement of enormous amount of water to cultivate. These two aquaporin-family proteins may be obtained by several mutational changes during evolution to acquire Al transport activities.

### Functional Analysis of HmVALT and HmPALT1 in Plants

**Figure 9:**
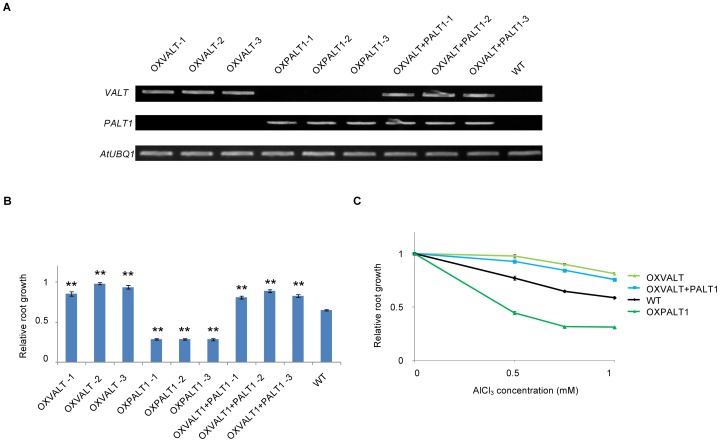
Effect of overexpression of *HmVALT* and/or *HmPALT1* in Arabidopsis. (**A**) The three independent lines of *HmVALT*- and/or *HmPALT1*-overexpressing Arabidopsis were grown in Al medium with 0 or 0.75 mM AlCl_3_ for 5 d. The mRNA expression of each gene was confirmed by RT-PCR. *HmVALT*, *HmPALT1* and *HmVALT* and *HmPALT1*-overexpressing plants were designated as OXVALT, OXPALT1, OXVALT+PALT1, respectively. (B) The root length of *HmVALT*- and/or *HmPALT1*-overexpressing Arabidopsis was measured with 0 or 0.75 mM AlCl_3_ for 5 d. The ratio of root length in plants treated with 0.75 mM AlCl_3_ to root length in plants without aluminum was displayed as the relative root length in the figure. Statistical significances were observed between the transgenic line and the non-transgenic line by Student's *t* test (**P<0.01 and *P<0.05). Error bars represent SE (each line was examined by n = 6). (C) The change of relative root length treated with various Al concentrations (0, 0.5, 0.75 and 1 mM). The each relative root length was shown as the average among three idependent lines of the same transgene. Error bars represent SE (n = 18).

To test the effects of the co-expression of these genes *in planta*, we prepared three independent transgenic Arabidopsis lines that was transformed with *HmVALT* and/or *HmPALT1*, and the Al-tolerance of the transformants were assessed through the quantification of root growth in seedlings (Figure 9). The each gene expression of each line was clarified by RT-PCR (Figure 9A). The average root length of the WT and each transformant expressing *HmVALT, HmPALT1,* and both *HmVALT* and *HmPALT1,* grown without Al for 5 days was 2.18 cm, 2.95 cm, 4.34 cm, and 2.34 cm, respectively. (Table S3). The Figure 9B, C showed the relative root length of each plant under Al-treatments as the length without Al to be 1.0.

The introduction of *HmVALT* significantly conferred Al-tolerance, and the introduction of *HmPALT1* significantly conferred Al-hypersensitivity from the result of relative root length treated with 0 or 0.75 mM AlCl_3_ (Figure 9B). Moreover, the latter phenomenon was compensated by the additional introduction of *HmVALT* (Figure 9B). When the Al-concentration in medium increased, the relative root lengths of WT plants decreased with dose-dependent manner (Figure 9C). In the *HmVALT*-overexpressing plants the relative root length was significantly increased, and in the *HmPALT1*-overexpressing plants that was significantly decreased compared with that of WT in each Al dose (Figure 9C). Furthermore, the relative root length in *HmVALT*- and *HmPALT1*-overexpressing Arabidopsis plants showed the recovery of the decrease in a dose dependent manner to the same degree as *HmVALT*-overexpressing ones (Figure 9C). These results could confirm the proposed mechanism that HmPALT1 transports Al into the cytosol, and then HmVALT secretes Al into the vacuoles (Figure 9B, C).

Without Al conditions, the absolute root length of the transformants expressing *HmPALT1* (4.34 cm) was longer than that of WT (2.18 cm) (Table S3). This phenomenon may be explained that HmPALT1 transports some essential elements in the external medium, which concentration is low and not a toxic level in the root cells but ultimately conferred growth improvement. Other NIP-family transporters, such as AtNIP5;1 and AtNIP6;1 [37], [38], could transport boric acid, one of essential element. These proteins are also reported to facilitate a bi-directional diffusion of As(III) [39]. Addition to this, iron-transporter protein, AtIRT1, belonging a member of the Zrt/Irt-like protein (ZIP) family has a broad specificity for divalent heavy metals and involving in accumulation of zinc, manganese, cobalt and cadmium under Fe-deficient conditions [49]. Therefore, it might be explained that HmPALT1 had a broad specificity and could import essential elements and toxic elements together into cytosol. To figure out the substrate specificity of HmPALT1 and HmVALT and the mechanism of the broad specificity is the next problem in this research.

In conclusion, we have identified two Al transporters in blue hydrangea sepals; one transporter is localized at the vacuolar membrane and the other at the plasma membrane. Both of the proteins belong to the aquaporin family and are involved in the expression of blue sepal color and Al tolerance in acidic soils. In blue colored hydrangea, the aluminum is transported from soil to sepals. In this absorption and distribution in the whole plant tissues aluminum transporter(s) should exist in roots, stems and leaves, together. But the HmPALT mRNA expression was only observed in sepals (Figure 7). This strongly indicates that other plasma membrane-localized aluminum transporter(s) should exist and work in these tissues. Furthermore, even in blue sepals, this unknown transporter may work together with HmPALT1 in order to transport a large amount of aluminum into the sepal cells. In this study we validated the efficiency of our strategy for functional gene research in non-model plants, such as hydrangea. Further studies regarding the regulation and long-distance transport of Al in hydrangea are in progress.

## Materials and Methods

### Plant Materials

The blue cultivar of *H. macrophylla* cv. Narumi blue was donated by Okumura Seikaen (Aichi, Japan) and was cultivated in an incubator under conditions of 12 h dark (15°C) and 12 h light (20,000 lux, 20°C).

To generate Arabidopsis plants overexpressing genes (VALT, PALT1 or VALT1 plus PALT1) under the control of the 35S promoter, the full-length cDNA of each gene was subcloned into a site of the multiple cloning site (MCS)1 (VALT or PALT1 for single gene overexpression) or MCS2 (PALT1 for overexpression of both genes) in the plasmid vector pRI201-AN (TaKaRa). VALT/pRI201, PALT1/pRI201 and VALT+PALT1/pRI201 were transformed into *Agrobacterium tumefaciens* GV3101 cells that subsequently were used to transfect Col-0 plants. We obtained three independent transgenic lines. T1 plants were selected on medium containing kanamycin (30 µg mL^−1^).

### RNA Extraction

The sepals from each growth stage were collected, and total RNA was extracted using an RNeasy Plant Mini Kit (QIAGEN) according to the manufacturer's protocol. Three stages with different sepal pigmentation were chosen (stage 1: no pigmentation in the sepals, stage 2: somewhat pigmented sepals and stage 3: fully pigmented sepals).

### Construction of a Full-length Enriched cDNA Library and Sequencing

Aliquots of total RNA from sepals at different stages (stages 1–3) were used for the construction of a full-length enriched cDNA library. The library was constructed by the biotinylated CAP trapper method and trehalose-thermoactivated reverse transcriptase, as described in previous reports [50], [51]. The resultant double-stranded cDNAs were ligated into a λFLC-III vector [52] by *in vivo* excision based on the Cre-loxP system. Approximately 12,000 clones were sequenced by the Sanger method using ABI 3730×l sequencers (Applied Biosystems). The Sanger sequence reads were assembled with Phred-Phrap software [53]. The overall accuracy of the sequence was estimated to have phred score of ≥20.

### Construction of DNA Microarray and Analysis

The DNA microarray was made as a 12,000 Combimatrix CustomArray (CombiMatrix Corporation). All probes for the array were designed using CombiMatrix ProbeWeaver ver. 1.0.10. One microgram of total RNA was reverse transcribed using the T7-oligo-dT primer, and double-stranded cDNAs (ds-cDNA) were generated using the Amino Allyl MessageAmp II aRNA Amplification Kit (Ambion). Antisense-RNA (aRNA) was amplified from ds-cDNA as a template, and labeled with amino allyl-modified aRNA by the Amino Allyl MessageAmp II aRNA Amplification Kit according to manufacturer's instructions. Five micrograms of the purified Cy5-aRNA sample was dissolved in 100 µl of hybridization solution (CombiMatrix CustomArray 12K Hybridization and Imaging Protocol: PTL006), and hybridization was conducted at 45°C for 16 h. The raw data were obtained from the images scanned using MicroarrayImager ver. 6.0.1 (CombiMatrix Corporation), and Microsoft Excel 2003 was used for background subtraction and median normalization. We employed simple fold-difference calculations using Microsoft Excel 2003 for each of the spot intensities between Stage 1:Stage 2, Stage 1:Stage 3, and Stage 2:Stage 3. From the sequence information, each gene function and subcellular localization was predicted by BLAST (NCBI) and WoLF PSORT (AIST). Among the genes with increased expression at stage 3, during which the Al contents of the sepals were increasing, candidate genes with potential substrate transporter functions and vacuole localization were selected. Furthermore, we narrowed the group of candidates to several genes that had more potential to transport aluminum into vacuoles. Yeast cells transformed with these genes were used in the Al transport activity tests described below.

### Aluminum Tolerance assay and Estimation of Aluminum Content in Yeast Cells

The cDNA fragment containing an entire ORF was amplified by PCR and inserted into the *Eco*R I site of pYES2 (Invitrogen) with an In-Fusion Advantage Cloning Kit with Cloning Enhancer (Clontech) using the primers shown in Table S2. The resulting plasmid was introduced into yeast cells. The yeast strains used in this study were the aluminum-sensitive mutant *Δhsp150* (*MATa his3Δ1 leu2Δ0 met15Δ0 ura3Δ0 YJL159w::KanMX4*) and BY4741 (*MATa his3Δ1 leu2Δ0 met15Δ0 ura3Δ0*). Transformants were selected on uracil-deficient medium and grown in synthetic complete (SC)-uracil yeast medium containing 2% glucose, 0.67% yeast nitrogen base without amino acids (Difco), the appropriate amino acids, and 2% agar at pH 5.8. For the aluminum-sensitivity test, yeast cells at mid-exponential phase were harvested and washed three times with ultra-pure water, and serial dilutions (0.1, 0.01, 0.001 and 0.0001) were spotted on LPP medium containing 1% agarose and 2% galactose (for induction of the GAL promoter) adjusted to pH 3.5. Each aluminum concentration is indicated as the added Al_2_(SO_4_)_3_ concentration. The cells were incubated for 3 d at 30°C. For the aluminum-sensitivity test in liquid medium, the pre-cultured yeast was adjusted to an OD_600_ value of 0.02 and then added to the media containing each aluminum concentration.

The Al concentration in yeast cells was measured in cells cultured for approximately 2 d in LPP medium. The cells were harvested by centrifugation and washed three times with ultra-pure water, and then the cell wall was digested with 200 U lyticase (Sigma) in buffer (50 mM Tris-HCl pH 7.5, 1.2 M sorbitol, 10 mM EDTA and 10 mM beta-mercaptoethanol) at 37°C overnight. The cells were collected by centrifugation, washed three times with 1.2 M sorbitol and then digested in 0.5% (w/v) HNO_3_. The aluminum content was measured by using Alumeasure (Nomura Chemical), which forms a fluorescent lumogallion-Al^3+^ complex that can be detected using HPLC, according to the manufacturer's instructions. A Develosil LAL (20 mmφ×100 mm) column was used, and the solvent Alumeasure ™R-3A (Nomura Chemical), containing 13% isopropanol, was used at a flow rate of 0.111 ml/min. The fluorescent signal (Ex = 505 nm, Em = 574 nm) was detected by FP-2020 Plus detector (Jasco). Each treatment was performed in triplicate.

### Particle Bombardment

*HmVALT* and *mCherry* were fused using the In-Fusion Advantage Cloning Kit with Cloning Enhancer (Clontech) and inserted into the transient vector pRI201-AN (TaKaRa) according to the manufacturer's instructions. This construct was designated pHmVALT-mCherry. The *HmPALT* cDNA fragment in pENTR-HmPALT1 was transferred to the transient expression vector, pGWmCherry, using Gateway technology according to the manufacturer's instructions (Invitrogen). This construct was designated pHmPALT1-mCherry. The transient expression vector pγTIP-GFP, which was used as a vacuolar membrane marker, was kindly provided by Ikuko Hara-Nishimura (Graduate School of Science, Kyoto University, Kyoto, Japan) [29]. The transient expression vector pPIP1A-GFP, which was used as a plasma membrane marker, was kindly provided by Anzu Minami (Graduate School of Science, Nagoya University, Japan). To examine the localization of HmVALT or HmPALT1, the plasmids were simultaneously introduced into onion epidermal cells using IDERA GIE-III (Tanaka), according to the manufacturer's instructions. The cells were bombarded with 1.0 µm gold particles by helium pressure. After incubation at 24°C for 21 h, fluorescence signals were detected using an LSM780-DUO-NLO confocal laser scanning microscope (Carl Zeiss).

### Quantitative RT-PCR Analysis

Total RNA was extracted from each tissue in hydrangea using TRIzol Reagent with the PureLink RNA Mini kit (Invitrogen). First-strand cDNA was synthesized from 2 µg of total RNA using SuperScript® III First-Strand Synthesis System for RT-PCR (Invitrogen). Real-time PCR was performed using a KAPA SYBR FAST qPCR kit (KAPABIOSYSTEMS) and ABI 7500 Fast Real-Time PCR system (Applied Biosystems) according to the manufacturer's instructions. The primer sets used for PCR were as follows: VALT, 5′-GGCCCTAGCAGAGTTCTTCTCT-3′ and 5′- AATGTAATGTTCCCACCAAGGA-3′; PALT1, 5′-ACCTGTAACTCCAGGGACTCCT-3′ and 5′-TATGAACTCAGCTCCCACCTTT-3′; 18SrRNA, 5′-GGAAGTTTGAGGCAATAACAGG-3′ and 5′-ATTGCAATGATCTATCCCCATC-3′.

### RT-PCR Analysis

To examine the expression patterns of *HmVALT* and *HmPALT1* in transgenic Arabidopsis plants, whole plants were excised and frozen in liquid nitrogen for RNA extraction. Total RNA was extracted using the RNeasy Plant Mini Kit (Qiagen). Ten nanograms of total RNA was used for the first -strand cDNA synthesis and subsequent PCR amplification using the SuperScript One-Step RT-PCR with platinum Taq kit (Invitrogen) according to the manufacturer's instructions. The nucleotide sequences of primers for RT-PCR of *HmVALT* and *HmPALT1* were 5′- GGCATGGCATTCAACAAGATTACA -3′ and 5′- TGTTTCCAATCCACCAGTGGCAAA -3′ and 5′- GCAAGTGCTTTGGTGTTGAA -3′ and 5′- TCTCGGAGCCTTGTGTCTTT -3′, respectively. For RT-PCR analysis of *HmVALT* and *HmPALT1* expression, cDNA synthesis and pre-denaturation was conducted by 1 cycle of 50°C for 30 min and 94°C for 2 min. The subsequent PCR consisted of 30 cycles at 94°C for 15 s, 50°C for 30 s and 72°C for 30 s, and a 5 min extension at 72°C. *AtUBQ1* used as internal control in transgenic Arabidopsis plants.

### Immunoblot Analysis

The synthetic peptides, C-GDSTHEQLPVTDY (amino acid residues of 240 to 252 of HmVALT) and C-GNTHDKPETAHSFRQ (amino acid residues of 290 to 304 of HmPALT1), were used to immunize rabbits to obtain antibodies against HmVALT and HmPALT1, respectively. The antisera obtained were purified through peptide affinity columns. The proteins of the hydrophobic fraction were extracted from hydrangea sepals at different stages (stages 1–3) using the Plant Hydrophobic Protein Extraction Kit (Sigma) according to the manufacturer's instructions. The fresh materials (approximately 0.2 g) of each tissue were frozen in liquid nitrogen and ground with a homogenizer. The protein content of the each fraction was measured using a Pierce 660-nm protein assay kit (Thermo Scientific) and bovine serum albumin as the standard. The proteins were separated by SDS–PAGE on a 12.5% polyacrylamide gel and then transferred to a Hybond-P PVDF membrane (GE Healthcare) using a semi-dry electroblotting system. Immunoreactive polypeptides were detected as the signals of Biotin-XX goat anti-rabbit IgG and Qdot625 streptavidin conjugate (Invitrogen) using a UV-transilluminator.

### Mutagenesis of *HmVALT* and *HmPALT1* and Expression in Yeast Cells

To create the nucleotide substitution at each selected amino acid residue of HmVALT and HmPALT1, we used a PrimeSTAR Mutagenesis Basal Kit (TaKaRa). The procedure was followed according to the manufacturer's instructions. The primer set was used as described in Table S2.

The *Δhsp150* mutant (for mutated HmVALT) or wild type (for mutated HmPALT1) yeasts were transformed with either pYES2, pYES 2GAL1-mutated HmVALT or pYES 2GAL1-mutated HmPALT1. The yeast cells were grown in SC medium lacking uracil with 2% glucose. After the cultures reached an optical density of 600 nm, the cells were washed three times with ultra-pure water, and serial dilutions (0.1, 0.01, 0.0001 and 0.00001) were spotted onto LPP medium plates with 2% galactose in the presence or absence of Al_2_(SO_4_)_3_. The cells were incubated for 4 d at 30°C.

### Aluminum Treatment for Arabidopsis Plants

The seeds of each line were soaked in one-sixth strength MS medium containing 0.3% (w/v) Gelrite (Wako) and kanamycin (for transgenic plants, 30 µg ml^−1^). The plates were stored at 4°C for 4 d in the dark to synchronize germination. After the dark treatment, the plates were placed vertically, and the seeds were cultivated in an incubator under the condition of 8 h dark and 16 h light (3,000 lux, 23°C) for 5 d. The transgenic plants resistant to kanamycin and col-0 plants were transferred to the plates one-sixth MS medium minus kanamycin and containing 0.3% (w/v) Gelrite and one of each concentration of AlCl_3_ at 0, 0.5, 0.75 and 1 mM, and the plates were placed vertically, and plants were grown for 5 d. The root length was measured with a ruler.

### Accession Numbers

Sequence data in this article can be found in Arabidopsis Genome Initiative or GenBank/EMBL databases under the following accession numbers: HmVALT, AB619645 and HmPALT1, AB619646. The microarray analysis data reported in this article are deposited in the GEO database under the accession numbers GSE26665 and GPL11530.

## Supporting Information

Table S1 **List of genes selected based on the microarray analyses.**
(XLS)

Table S2 **Primers used in this work.**
(XLS)

Table S3 **The absolute root length without aluminum.**
(XLS)
